# Evaluation and Clinical Validation of Guanidine-Based Inactivation Transport Medium for Preservation of SARS-CoV-2

**DOI:** 10.1155/2022/1677621

**Published:** 2022-07-21

**Authors:** Hesti L. Wiraswati, Shabarni Gaffar, Savira Ekawardhani, Nisa Fauziah, Fedri R. Rinawan, Leonardus Widyatmoko, Amila Laelalugina, Annissa R. Arimdayu, Tri Kusniati, Clarisa D. Andari, Lia Faridah

**Affiliations:** ^1^Parasitology Laboratory, Advanced Biomedical Laboratory, Faculty of Medicine, Universitas Padjadjaran, Bandung, Indonesia; ^2^C.29 Laboratory, Faculty of Medicine, Universitas Padjadjaran, Bandung, Indonesia; ^3^Parasitology Division, Department of Biomedical Science, Faculty of Medicine, Universitas Padjadjaran, Bandung, Indonesia; ^4^Infection Study Center, Faculty of Medicine, Universitas Padjadjaran, Bandung, Indonesia; ^5^Department of Chemistry, Faculty of Mathematics and Natural Sciences, Universitas Padjadjaran, Bandung, Indonesia; ^6^Research Center for Biotechnology and Bioinformatics, Universitas Padjadjaran, Bandung, Indonesia; ^7^Department of Public Health, Faculty of Medicine, Universitas Padjadjaran, Bandung, Indonesia; ^8^Clinical Microbiology Laboratory, Santosa Hospital Bandung Central, Bandung, Indonesia

## Abstract

WHO declared the outbreak of COVID-19, caused by SARS-CoV-2, a pandemic in March 2020. More than 223 million cases and approximately 4.6 million deaths have been confirmed. Early diagnosis and immediate treatment became a priority during this pandemic. However, COVID-19 diagnostic testing resources are limited, especially early in the pandemic. Apart from being limited, the COVID-19 diagnostic tests using reverse transcription polymerase chain reaction (RT-PCR) have encountered storage, transportation, and safety issues. These problems are mainly experienced by developing poor countries, countries in the equatorial region, and archipelagic countries. VITPAD® is a guanidine-based inactivation transport medium (ITM) formulated to maintain the RNA quality of SARS-CoV-2 during transportation without cold chains. This study, conducted from September 2020 to March 2021, performed clinical validation of VITPAD® by comparing its performance with a globally commercially available ITM from the NEST brand. Its stability at room temperature, safety, and resistance at high temperatures was also tested using RT-PCR analysis. VITPAD® can reduce the infectious nature of the specimen, preserve the SARS-CoV-2 for 18 days at an ambient temperature, and resist high temperatures (40°C for 3 hours). A guanidine-based transport medium, such as VITPAD®, is compatible and recommended for RT-PCR-based molecular diagnosis of COVID-19.

## 1. Introduction

COVID-19 (coronavirus disease of 2019), caused by the SARS-CoV-2 (severe acute respiratory syndrome coronavirus 2), spread worldwide, being declared a pandemic by WHO (World Health Organization) in March 2020. As of September 2021, WHO has reported more than 223 million SARS-CoV-2 cases globally, with approximately 4.6 million deaths occurring in more than 230 WHO regions (regional organizational groupings by WHO, based on geographical terms but are not synonymous with geographical areas) [1].

Early diagnosis and immediate treatment of COVID-19 became a priority in order to reduce the spread of disease and break the chain of transmission [2, 3]. Diagnostic testing for the presence of SARS-CoV-2 viral RNA (ribonucleic acid) typically relies on detecting the virus through nucleic acid amplification or antigen identification. Following collection of a patient test sample, it is typically stored in a transport medium before transport to a testing facility [4–6].

The increasing demand for early diagnosis has placed significant demand on supply chains, including in the availability of testing equipment, assay components, swabs, and transport mediums. Therefore, there is a shortage of diagnostic resources. This shortage has become limiting for COVID-19 diagnosis testing [6–9]. As a result, delays in diagnosis and the rationing of diagnostic testing have been seen [5, 10–13].

One source of this shortage is the limited resource available recommended by WHO and the Centre for Disease Control and Prevention (CDC). For COVID-19 diagnostic testing, WHO and the CDC recommend nasal or pharyngeal swabs that are stored in a viral transport medium (VTM) and tested using a nucleic acid amplification test (NAAT) [5, 9, 14–16]. Unfortunately, NAAT for SARS-CoV-2 detection has several limitations in terms of sensitivity among its various protocols [17, 18]. There is also the possibility of a false negative of the RT‐PCR test due to prolonged nucleic acid conversion (the period from the date of symptoms onset to the date of first negative RT-PCR test result) [19], changes in diagnostic accuracy over the disease course, and precarious availability of test materials [20]. Nevertheless, NAAT remains the gold standard globally [21] and is a better reference for developing alternative methods [22]. NAAT is also used as a rule for international travel by authorities [23–25]. It is urgently needed to help identify new variants of COVID-19 by PCR-SGTF (S-gene target failure) or whole-genome sequencing (WGS) [26–28].

Diagnostic test laboratories have used several alternative methods to NAAT [10, 16, 29–39]. However, all alternative methods still require the sample collection step and, therefore, require a swab and transport medium. The sample collection step includes various processes, such as specimen collection, packaging, storage, and transportation. Therefore, improper sample collection could affect the overall assay performance, the quality of the specimen, and the accuracy of COVID-19 diagnosis [15, 40, 41].

Some researchers have discussed alternatives to swabs [42] and transport medium [12, 13, 43, 44]. For example, Panpradist et al. (2020) recommended dry swabs to eliminate the need for a transport medium [45]. However, dry swabs have been found to have a false-negative rate of 47% [46]. Therefore, the transport medium is crucial for reliable COVID-19 diagnostic testing. Moreover, Scheier et al. (2021) did not detect any SARS-CoV-2 contamination of open sampling transport medium tubes during nasopharyngeal swab (NPS) sampling [47]. Healthcare workers could avoid contamination during NPS sampling by following biosafety protocols [6, 38, 48–50].

Many transport media are suitable for preserving viruses, including VTM or saline [51]. For example, a VTM based on culture medium is recommended by the CDC and WHO. However, it should be kept refrigerated at 2–8°C or frozen at −70°C or below (for storage more than 72 hours) [15, 41]. The transportation of these VTM is hard to fulfill in the development of poor countries where the electricity or cold-chain system is distributed not evenly. It is also difficult for the countries in the equatorial region and archipelagic countries, which need assurance and biosafety of the COVID-19 sample transportation.

Guanidinium thiocyanate or guanidinium hydrochloride has shown to inactivate SARS-CoV-2 and can be used for RT-PCR applications [52–54] since inactivation reagents were effective at reducing viral titers [55]. It was suggested to be used as part of SARS-CoV-2 NAAT at high-risk locations (schools, workplaces, prisons, skilled nursing facilities, homeless shelters, etc.) [52]. In this research, VITPAD®, a guanidine-based inactivation transport media (ITM) formulated to maintain the RNA quality of SARS-CoV-2 during transportation without cold chains, was evaluated. It claims to reduce the infectious nature of the sample while maintaining the quality of the specimen. VITPAD® ITM is a licensed nasopharyngeal specimen collection swab and medium storage from the Indonesian Ministry of Health (AKD 10302120146, PDKI BRM2054A).

## 2. Materials and Methods

### 2.1. Preparation

Inactivation preservation of SARS-CoV-2 was carried out from September 2020 to March 2021 at the Parasitology Laboratory, Advanced Biomedical Laboratory of the Universitas Padjadjaran. The preservation used was the adaptation of WHO guidance [56] where the viral transport medium was substituted with the inactivation transport medium. This study evaluated VITPAD® (Indonesian Ministry of Health AKD 10302120146, PDKI BRM2054A). VITPAD® is a domestically commercially available VTM. It contains the inactivating ingredient guanidine. VITPAD® consists of an NPS, storage tube (cryotube), and 2 mL of guanidine-based inactivation transport media.

VITPAD® ITM was compared with globally commercially available ITM from the NEST brand. Its stability at room temperature, safety, and performance at high temperatures were also evaluated. The tests and reagents are described below (see Table 1).

### 2.2. SARS-CoV-2 RNA Detection

We conducted NPS sampling of 99 subjects for the COVID-19 diagnosis test. Each subject was sampled 2 times: the first NPS was stored in VITPAD® ITM, and the second NPS was stored in NEST ITM. Samples were extracted, and viral RNA (gene targets: ORF1ab, N-gene, and E-gene) was measured by RT-PCR following the protocol specified by the RT-PCR kit used (Table 1).

To test the stability of VITPAD® ITM at room temperature (±25°C), NPS sampling of 30 subjects of the COVID-19 diagnosis test was stored in VITPAD®. It was aliquoted into 300-*µ*L tubes. Each aliquot sample was stored at room temperature (±25°C). Viral RNA was measured after set periods had lapsed (0 days, 4 days, 8 days, 12 days, and 18 days) following the protocol specified by the RT-PCR kit used (Table 1).

The safety of VITPAD® ITM was tested by comparing NPS sampling of 38 subjects of the COVID-19 diagnosis test that was extracted with a lysis buffer and without a lysis buffer. Viral RNA was then detected by RT-PCR following the protocol specified by the RT-PCR kit used (Table 1).

For the resistance of VITPAD® ITM at high temperatures, NPS sampling of 12 subjects of the COVID-19 diagnosis test was aliquoted into 250-*µ*L tubes. Each aliquot sample was incubated at 40°C for three hours. This temperature was chosen as it is the average estimated highest ambient temperature in Indonesia, while 3 hours was chosen by the average estimated travel time between Indonesian cities. The viral RNA was measured by RT-PCR following the protocol specified by the RT-PCR kit used (Table 1) and compared with control samples that were stored at room temperature (±25°C).

### 2.3. Statistical Analysis

For clinical validation, the internal control cycle threshold value (IC CT value) was analyzed by using the receiver operating characteristic (ROC) curve analysis approach. Receiver operating characteristic (ROC) curves were analyzed by using licensed IBM SPSS Version 26. Ordinary one-way ANOVA with Bonferroni and Šídák multiple comparison tests was applied to calculate and compare *P*-values for VITPAD® ITM stability test at room temperature (±25°C) for 4, 8, 12, and 18 days. An unpaired *t*-test was used for the analysis of the VITPAD® ITM safety test and the resistance test at 40°C. The distribution of the data was tested by the D'Agostino–Pearson normality test. *P*-value <0.05 is considered statistically significant. Analysis of these data and graphing were done by GraphPad Prism 9.0.0 for Windows.

## 3. Results

### 3.1. Comparison between VITPAD® ITM and NEST ITM

Out of the 99 samples tested, VITPAD® ITM positive rate is 19.2%. Meanwhile, NEST ITM positive rate is 15.2% with an invalid result of 1% (*P*=0.572, Fisher's exact test. The positive rate results are not affected by the type of ITM brand). The data are displayed below (see Table 2).

**Figure 1:**
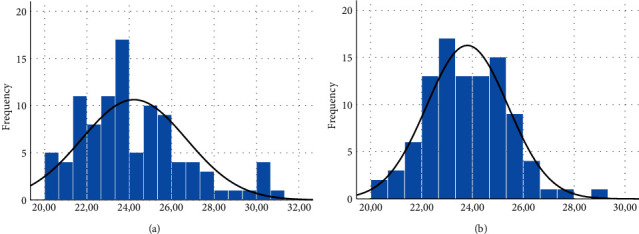
Calculation of the internal control cycle threshold value (IC CT value) of the Sansure reagent. (a) Samples stored in VITPAD® ITM: mean IC CT value = 24.22, standard deviation = 2.476, and number of sample = 99; (b) samples stored in NEST ITM mean IC CT value = 23.80, standard deviation = 1.601, and number of sample = 98.

Figure 1 describes the internal control cycle threshold value (IC CT value) of the Sansure reagent of VITPAD® ITM. Samples were clustered to the left side, near zero (skewness value = 0.805; median = 23.74; mode = 20.62; kurtosis = 0.409; Shapiro–Wilk = 0.001). The IC CT values of the NEST ITM samples were clustered in the middle (skewness value = 0.291; median = 23.745; mode = 23; kurtosis = 0.397; Shapiro–Wilk = 0.579). The left-clustered data of the VITPAD® ITM mean that the data had lower IC CT values. Therefore, the VITPAD® ITM is more stable regarding retaining samples, namely, the human epithelium contained in the IC of the Sansure reagent. The implication of this is that more RNA could be calculated for the COVID-19 diagnostic test, thereby reducing the risk of obtaining invalid results.

**Figure 2:**
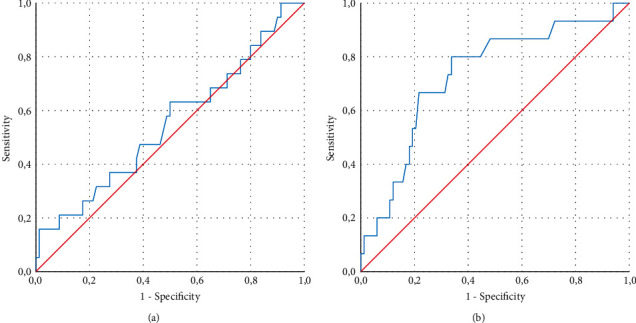
Receiver operating characteristic (ROC) curve. (a) Samples stored in VITPAD® ITM; (b) samples stored in NEST ITM.

A ROC curve analysis approach was used for calculating the IC CT value of the Sansure reagent. This ensured that the performance of each ITM regarding maintaining sample stability was not associated with the cutoff of positive and negative values of the SARS-CoV-2 target gene. As seen in Figure 2, the area under the curve was not significant for the VITPAD® ITM (*P*=0.502), while on the NEST ITM, it was significant (*P*=0.004). In this case, a nonsignificant result means that the VITPAD® ITM is not associated with the cutoff positive and negative values of the SARS-CoV-2 target gene.

### 3.2. VITPAD® ITM Stability at Room Temperature (±25°C)

VITPAD® ITM maintained the CT value of all the target genes (ORF1ab, N-gene, and E-gene) at room temperature (±25°C). Using RT-PCR, samples were tested for SARS-CoV-2 RNA after 0, 4, 8, 12, and 18 days. One-way ANOVA test results found that the *P*-value for each target gene was more than 0.05 and, therefore, not statistically significant (see Table 3).

**Figure 3:**
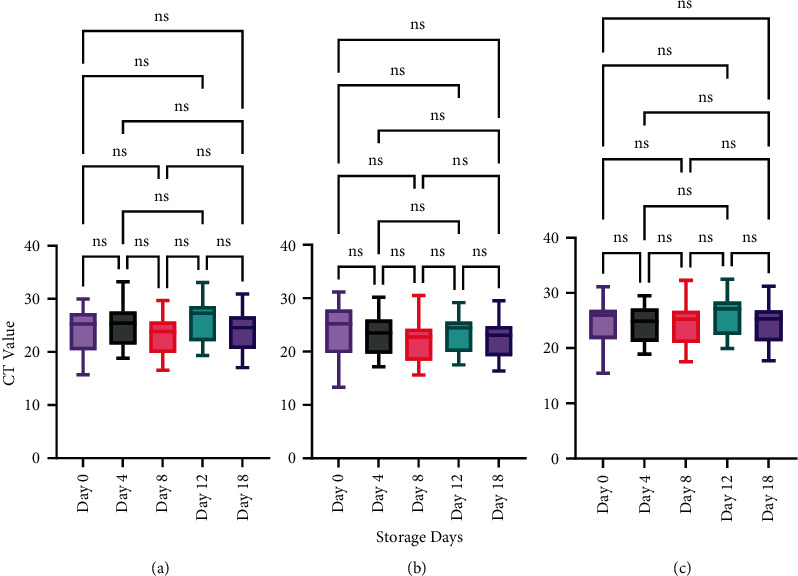
CT value of target genes for the stability test at room temperature (±25°C). CT values of samples stored for 0 days, 4 days, 8 days, 12 days, and 18 days at room temperature (±25°C): (a) ORF1ab; (b) N-gene; and (c) E-gene. ns—not statistically significant. *P*-value >0.05 is considered not statistically significant.

The ANOVA tests were followed by Bonferroni and Šídák tests to compare the difference between each storage day (multiple comparisons). Bonferroni and Šídák tests were also not statistically significant for all the target genes (see Figure 3). VITPAD® ITM can maintain the sample at room temperature for 18 days.

### 3.3. Safety Test of VITPAD® ITM

**Figure 4:**
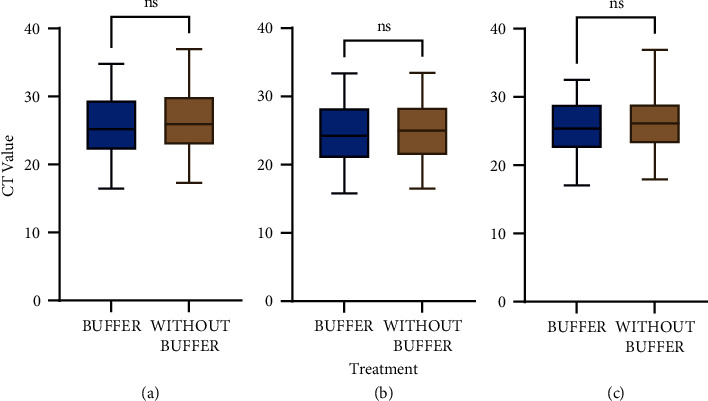
CT value of target genes for the safety test. CT values of COVID-19 samples stored in VITPAD® ITM extracted with and without the lysis buffer: (a) ORF1ab; (b) N-gene; and (c) E-gene. ns—not statistically significant. *P*-value >0.05 is considered not statistically significant.

There was no significant difference between the CT values of COVID-19 target genes in samples stored in the VITPAD® ITM (*P* > 0.05, unpaired *t*-test). Therefore, the extraction procedure of COVID-19 samples stored in VITPAD® ITM could be performed with or without the use of a lysis buffer prior to performing RT-PCR, as the CT values will not differ (see Figure 4).

### 3.4. VITPAD® ITM Performance at 40°C Temperature

**Figure 5:**
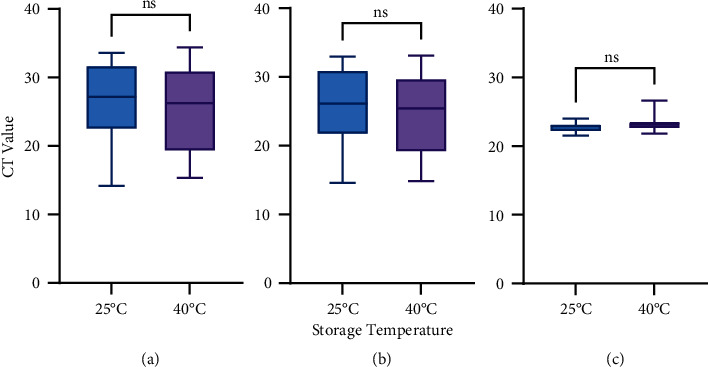
CT value of target genes for the resistance test. CT values of COVID-19 samples stored in VITPAD® ITM at room temperature (±25°C) and 40°C (for 3 hours): (a) ORF1ab; (b) N-gene; and (c) Internal control. ns—not statistically significant. *P*-value >0.05 is considered not statistically significant.

There was no significant difference between the CT values of the COVID-19 target genes in the samples stored for 3 hours in the VITPAD® ITM at ±25°C or 40^o^C (*P* > 0.05, unpaired *t*-test). Therefore, COVID-19 samples could be stored in the VITPAD® ITM at 40^o^C for 3 hours without impacting the CT value of the diagnostic test results (see Figure 5).

## 4. Discussion

COVID-19 diagnostic testing requires a reliable transport medium. Specifically, it requires a viral medium that remains stable in the environment or when exposed to high temperatures and remains safe from interference during the storage or sample transfer process. In 2020, Garnett et al. [42] and van Bockel et al. [50] reported a variety of swabs and transport mediums suitable for SARS-CoV-2 testing. However, the number of VTMs is still limited in many countries. This problem led to the development of a domestic transport medium (VITPAD® ITM) for COVID-19 diagnostic testing in Indonesia. This transport medium is equipped with nylon NPSs, which are six times more resistant than Dacron swabs [40], and was recommended by the CDC in their recent guidelines for SARS-CoV-2 diagnosis [57].

The VITPAD® ITM reagent is a buffer, solution-based medium that enables samples to be stored at room temperature. For health facilities that have a limited number of cold storage spaces, this will be beneficial. The clinical evaluation showed that the VITPAD® ITM produced similar results when compared to the NEST ITM. However, surprisingly, the majority of IC CT values regarding the Sansure reagent of the VITPAD® ITM were lower. Therefore, the VITPAD® ITM has more stability regarding retaining the sample. This result aligns with the findings of Nairz et al. (2021) [58], who stated that an in-house swab system was superior to most commercially available sets, indicated by definitively lower CT values of viral genes. This report shows that the VITPAD® ITM can effectively maintain the stability of nucleic acids in swab samples during transportation from the sampling site to a COVID-19 RT-PCR laboratory.

Transportation of samples is a challenge for health facilities located at significant distances away from RT-PCR laboratories. Transportation in such cases requires a cooling system for sample collection and delivery. Nucleic acids in biological samples are susceptible to degradation if they are not stored between 2 and 8°C. Long-term storage (more than 72 hours) for further analysis, that is, whole-genome sequencing (WGS), viral culture, or diagnostic purposes, requires a freezer (70°C or below).

Stability tests indicated that the VITPAD® ITM is able to maintain nucleic acid stability at room temperature for a storage period of up to 18 days. This would be beneficial during sample transfer, as sample handling would be simplified as the transfer process could occur without a cool box, saving space, and limiting the risk of sample damage. This could also be beneficial in the event that an RT-PCR laboratory becomes overloaded with samples, as the samples could be stored without the need for cold storage facilities. Moreover, the number of health facilities is higher than the number of COVID-19 RT-PCR laboratories.

Without the need for cold storage facilities, the RT-PCR laboratory could store more samples in their existing storage rooms. The sampling processes that occur at health facilities determine whether a nucleic acid sample can be analyzed properly in a COVID-19 RT-PCR laboratory. The VITPAD® ITM would be able to increase the efficiency of a mass screening program, particularly if access to an icebox, fridge, or freezer is unreliable or nonexistent at the sampling site.

While genetic materials deteriorate more rapidly at higher temperatures [59] and the SARS-CoV-2 is sensitive to heat [60], the use of the VITPAD® ITM could mitigate concerns of SARS-CoV-2 RNA degradation due to heat exposure. The performance tests showed that the diagnostic test results were stable after 3 hours of incubation at 40°C. Therefore, the VITPAD® ITM can transport samples when temperatures are high, above room temperature (±25°C). This is important, as the temperature during the summer months often reaches temperatures of more than 30°C in many countries.

In Indonesia, data from the Meteorological, Climatological, and Geophysical Agency stated that the maximum temperature in Indonesia is trending toward 40^o^C [61]. The VITPAD® ITM could simplify the transportation and collection of COVID-19 specimens in a remote or isolated area. Furthermore, the VITPAD® ITM is safe, as the virus inside the transport medium is in the form of genetic material (RNA) and, therefore, may be less infectious than in its viral form. Based on this, it is highly recommended that the VITPAD® ITM be used for the PCR-based molecular diagnosis of COVID-19.

With the limited number of samples and variation of the machines and reagents due to its limitations and availability throughout the research, we ensure that every comparative analysis between each variable in each type of test (comparison between VITPAD® ITM and NEST ITM; stability of VITPAD® ITM at room temperature (±25^o^C); safety test of VITPAD® ITM; and VITPAD® ITM high-temperature resistance (40^o^C)) carried out using the same machines and consumables (Table 1). This was done because various RT-PCR protocols may not have a similar analytical or clinical sensitivity and specificity even when used for the same COVID-19 clinical sample [18]. Therefore, we ensured this research use of the same consumables and RT-PCR protocols in each variable in each test type.

## 5. Conclusions

The VITPAD® ITM can be used as a transport medium for diagnostic tests of COVID-19. It can preserve the SARS-CoV-2 for 18 days at room temperature (±25°C). It maintains sample stability even after 3 hours of incubation at 40^o^C. The VITPAD® ITM reduces the potential of biohazard events occurring, as most of the samples are in the form of RNA. Therefore, this transport medium can effectively facilitate the transportation and collection of COVID-19 specimens, particularly for remote or isolated areas in Indonesia.

## Figures and Tables

**Table 1:** Reagents preparation.

	Comparison between VITPAD® ITM and NEST ITM	Stability at room temperature (±25°C)	Safety test	High temperature resistance (40°C)
*Extraction Machine*				
KingFisher™ Flex Purification System	✓	✓	✓	✓

*Extraction reagents*				
Sansure Biotech Novel Coronavirus (2019-nCoV) Nucleic Acid Diagnostic Kit (PCR-Fluorescence Probing)	✓			
MagEx RNA Extraction Kit		✓	✓	
MGI SARS-CoV-2 Automated Extraction Solutions				✓

*RT-PCR machine*				
Roche LightCycler® 480 Real-Time PCR System	✓			
Agilent AriaMx Real-Time PCR System		✓	✓	
LightCycler® 480 Real-Time PCR System				✓

*RT-PCR reagents*				
Sansure Biotech Novel Coronavirus (2019-nCoV) Nucleic Acid Diagnostic Kit (PCR-Fluorescence Probing)	✓			
Fosun COVID-19 RT-PCR Detection Kit		✓	✓	
BioPerfectus Technologies COVID-19 Coronavirus Real Time PCR Kit				✓

^*∗*^The variation of the machines and reagents was due to its limitation and availability throughout the research.

**Table 2:** COVID-19 diagnostic test results of samples stored in VITPAD® and NEST ITM.

Diagnostic test results	VITPAD® ITM	NEST ITM
Positive SARS-CoV-2 *n* (%)	19 (19.2%)	15 (15.2%)
Negative SARS-CoV-2 *n* (%)	80 (80.8%)	83 (83.8%)
Invalid	—	1 (1%)

Total	99 (100%)	99 (100%)

**Table 3:** One-way ANOVA results.

Target gene	*F*	*P*-value	*R* squared
ORF1ab	1.891	0.138	0.070
*N*-gene	0.789	0.504	0.030
*E*-gene	1.286	0.286	0.049

^*∗*^The result was considered significant when the *P*-value was less than 0.05.

## Data Availability

All data generated or analyzed during this study are included within the article and in supplementary information files.

## Supplementary Materials

Supplementary Materials Additional file APPENDIX: (I) Appendix 1. RT-PCR results of COVID-19 samples stored in VITPAD® ITM at room temperature (±25°C) for different storage days. (II) Appendix 2. RT-PCR results of COVID-19 samples in VITPAD® ITM, extracted with and without lysis buffer. (III) Appendix 3. RT-PCR results of COVID-19 samples stored in VITPAD® ITM, incubated at 40°C for 3 hours. (IV) Appendix 4. Receiver operating characteristic (ROC) curve statistical analysis. (V) Appendix 5. *t*-test for the safety of VITPAD® ITM. (VI) Appendix 6. *t*-test for the resistance of VITPAD® ITM.

## References

[1] World Health Organization (2021). WHO Coronavirus (COVID-19) Dashboard. https://covid19.who.int/table.

[2] Tomo S., Karli S., Dharmalingam K., Yadav D., Sharma P. (2020). The clinical laboratory: a key player in diagnosis and management of COVID-19. *EJIFCC*.

[3] Tu Y. P., O’Leary T. J. (2020). Testing for severe acute respiratory syndrome-coronavirus 2: challenges in getting good specimens, choosing the right test, and interpreting the results. *Critical Care Medicine*.

[4] Esbin M. N., Whitney O. N., Chong S., Maurer A., Darzacq X., Tjian R. (2020). Overcoming the bottleneck to widespread testing: a rapid review of nucleic acid testing approaches for COVID-19 detection. *RNA*.

[5] Ravi N., Cortade D. L., Ng E., Wang S. X. (2020). Diagnostics for SARS-CoV-2 detection: a comprehensive review of the FDA-EUA COVID-19 testing landscape. *Biosensors and Bioelectronics*.

[6] McAuley J., Fraser C., Paraskeva E. (2021). Optimal preparation of SARS-CoV-2 viral transport medium for culture. *Virology Journal*.

[7] Mascuch S. J., Fakhretaha-Aval S., Bowman J. C. (2020). A blueprint for academic laboratories to produce SARS-CoV-2 quantitative RT-PCR test kits. *Journal of Biological Chemistry*.

[8] Shetty O., Gurav M., Bapat P. (2021). COVID 19 pandemic testing time - crisis or opportunity in disguise for India?. *Seminars in Oncology*.

[9] Younes N., Al-Sadeq D. W., Al-Jighefee H. (2020). Challenges in laboratory diagnosis of the novel coronavirus SARS-CoV-2. *Viruses*.

[10] Calvez R., Taylor A., Calvo-Bado L., Fraser D., Fink C. G. (2020). Molecular detection of SARS-CoV-2 using a reagent-free approach. *PLoS One*.

[11] Morehouse Z. P., Proctor C. M., Ryan G. L., Nash R. J. (2020). A novel two-step, direct-to-PCR method for virus detection off swabs using human coronavirus 229E. *Virology Journal*.

[12] Radbel J., Jagpal S., Roy J. (2020). Detection of severe acute respiratory syndrome coronavirus 2 (SARS-CoV-2) is comparable in clinical samples preserved in saline or viral transport medium. *Journal of Molecular Diagnostics*.

[13] Rogers A. A., Baumann R. E., Borillo G. A. (2020). Evaluation of transport media and specimen transport conditions for the detection of SARS-CoV-2 by use of real-time reverse transcription-PCR. *Journal of Clinical Microbiology*.

[14] Feng W., Newbigging A. M., Le C. (2020). Molecular diagnosis of COVID-19: challenges and research needs. *Analytical Chemistry*.

[15] Kilic T., Weissleder R., Lee H. (2020). Molecular and immunological diagnostic tests of COVID-19: current status and challenges. *iScience*.

[16] Alaifan T., Altamimi A., Obeid D., Alshehri T., Almatrrouk S., Albarrag A. (2021). SARS-CoV-2 direct real-time polymerase chain reaction testing in laboratories with shortage challenges. *Future Virology*.

[17] Dramé M., Tabue Teguo M., Proye E. (2020). Should RT-PCR be considered a gold standard in the diagnosis of COVID-19?. *Journal of Medical Virology*.

[18] Sule W. F., Oluwayelu D. O. (2020). Real-time RT-PCR for COVID-19 diagnosis: challenges and prospects. *Pan African Medical Journal*.

[19] Xiao A. T., Tong Y. X., Zhang S. (2020 Oct). False negative of RT-PCR and prolonged nucleic acid conversion in COVID-19: rather than recurrence. *Journal of Medical Virology*.

[20] Sidiq Z., Hanif M., Dwivedi K. K., Chopra K. K. (2020). Benefits and limitations of serological assays in COVID-19 infection. *Indian Journal of Tuberculosis*.

[21] WHO (2021). WHO provides one million antigen-detecting rapid diagnostic test kits to accelerate COVID-19 testing in Indonesia. https://www.who.int/indonesia/news/detail/17-03-2021-who-provides-one-million-antigen-detecting-rapid-diagnostic-test-kits-to-accelerate-covid-19-testing-in-indonesia.

[22] Waller J. V., Kaur P., Tucker A. (2020). Diagnostic tools for coronavirus disease (COVID-19): comparing CT and RT-PCR viral nucleic acid testing. *American Journal of Roentgenology*.

[23] Consulate general of the republic of Indonesia (2021). Update: Indonesia travel restrictions. https://kemlu.go.id/losangeles/en/news/11727/update-indonesia-travel-restrictions.

[24] Ministry of the Interior of France (2022). COVID-19: international travel. https://www.interieur.gouv.fr/covid-19-international-travel.

[25] Centers for Disease Control and Prevention (2022). Required testing before air travel to the US. https://www.cdc.gov/coronavirus/2019-ncov/travelers/testing-international-air-travelers.html.

[26] Coolen J. P. M., Wolters F., Tostmann A. (2021). SARS-CoV-2 whole-genome sequencing using reverse complement PCR: for easy, fast and accurate outbreak and variant analysis. *Journal of Clinical Virology*.

[27] Wolter N., Jassat W., Walaza S. (2022). Early assessment of the clinical severity of the SARS-CoV-2 omicron variant in South Africa: a data linkage study. *The Lancet*.

[28] Tseng H. F., Ackerson B. K., Luo Y. (2022). Effectiveness of mRNA-1273 against SARS-CoV-2 omicron and delta variants. *Nature Medicine*.

[29] Adams N. M., Leelawong M., Benton A., Quinn C., Haselton F. R., Schmitz J. E. (2021). COVID-19 diagnostics for resource-limited settings: evaluation of “unextracted” qRT-PCR. *Journal of Medical Virology*.

[30] Agarwal R., Gupta E., Dubey S. (2021). Pooled nasopharyngeal swab collection in a single vial for the diagnosis of SARS CoV-2 infection: an effective cost saving method. *Indian Journal of Medical Microbiology*.

[31] Andryukov B. G., Besednova N. N., Kuznetsova T. A., Fedyanina L. N. (2021). Laboratory-based resources for COVID-19 diagnostics: traditional tools and novel technologies. a perspective of personalized medicine. *Journal of Personalized Medicine*.

[32] Arumugam A., Faron M. L., Yu P., Markham C., Wu M., Wong S. (2020). A rapid SARS-CoV-2 RT-PCR assay for low resource settings. *Diagnostics*.

[33] Barza R., Patel P., Sabatini L., Singh K. (2020). Use of a simplified sample processing step without RNA extraction for direct SARS-CoV-2 RT-PCR detection. *Journal of Clinical Virology*.

[34] Hasan M. R., Mirza F., Al-Hail H. (2020). Detection of SARS-CoV-2 RNA by direct RT-qPCR on nasopharyngeal specimens without extraction of viral RNA. *PLoS One*.

[35] Kobayashi R., Murai R., Asanuma K., Fujiya Y., Takahashi S. (2021). Evaluating a novel, highly sensitive, and quantitative reagent for detecting SARS-CoV-2 antigen. *Journal of Infection and Chemotherapy*.

[36] Lim K. L., Johari N. A., Wong S. T. (2020). A novel strategy for community screening of SARS-CoV-2 (COVID-19): sample pooling method. *PLoS One*.

[37] Qian J., Boswell S. A., Chidley C. (2020). An enhanced isothermal amplification assay for viral detection. *Nature Communications*.

[38] Yamamoto K., Suzuki M., Yamada G. (2021). Utility of the antigen test for coronavirus disease 2019: factors influencing the prediction of the possibility of disease transmission. *International Journal of Infectious Diseases*.

[39] Wei S., Kohl E., Djandji A. (2021). Direct diagnostic testing of SARS-CoV-2 without the need for prior RNA extraction. *Scientific Reports*.

[40] Bidkar V., Selvaraj K., Mishra M., Shete V., Sajjanar A. (2021). A comparison of swab types on sample adequacy, suspects comfort and provider preference in COVID-19. *American Journal of Otolaryngology*.

[41] Shrestha L. B., Pokharel K. (2020). Standard operating procedure for specimen collection, packaging and transport for diagnosis of SARS-COV-2. *JNMA; Journal of the Nepal Medical Association*.

[42] Garnett L., Bello A., Tran K. N. (2020). Comparison analysis of different swabs and transport mediums suitable for SARS-CoV-2 testing following shortages. *Journal of Virological Methods*.

[43] Perchetti G. A., Huang M. L., Peddu V., Jerome K. R., Greninger A. L. (2020). Stability of SARS-CoV-2 in phosphate-buffered saline for molecular detection. *Journal of Clinical Microbiology*.

[44] Rodino K. G., Espy M. J., Buckwalter S. P. (2020). Evaluation of saline, phosphate-buffered saline, and minimum essential medium as potential alternatives to viral transport media for SARS-CoV-2 testing. *Journal of Clinical Microbiology*.

[45] Panpradist N., Wang Q., Ruth P. S. (2021). Simpler and faster COVID-19 testing: strategies to streamline SARS-CoV-2 molecular assays. *EBioMedicine*.

[46] Thwe P. M., Ren P. (2020). How many are we missing with ID NOW COVID-19 assay using direct nasopharyngeal swabs? Findings from a mid-sized academic hospital clinical microbiology laboratory. *Diagnostic Microbiology and Infectious Disease*.

[47] Scheier T., Shah C., Huber M. (2021). Do we cause false positives? An experimental series on droplet or airborne SARS-CoV-2 contamination of sampling tubes during swab collection in a test center. *Antimicrobial Resistance and Infection Control*.

[48] Islam K. U., Iqbal J. (2020). An update on molecular diagnostics for COVID-19. *Frontiers in Cellular and Infection Microbiology*.

[49] Karthik K., Aravindh Babu R. P., Dhama K. (2020). Biosafety concerns during the collection, transportation, and processing of COVID-19 samples for diagnosis. *Archives of Medical Research*.

[50] van Bockel D., Munier C. M. L., Turville S. (2020). Evaluation of commercially available viral transport medium (VTM) for SARS-CoV-2 inactivation and use in point-of-care (POC) testing. *Viruses*.

[51] Druce J., Garcia K., Tran T., Papadakis G., Birch C. (2012). Evaluation of swabs, transport media, and specimen transport conditions for optimal detection of viruses by PCR. *Journal of Clinical Microbiology*.

[52] Banik S., Saibire K., Suryavanshi S. (2021). Inactivation of SARS-CoV-2 virus in saliva using a guanidium based transport medium suitable for RT-PCR diagnostic assays. *PLoS One*.

[53] Roberts P. L., Lloyd D. (2007). Virus inactivation by protein denaturants used in affinity chromatography. *Biologicals*.

[54] Weidner L., Laner-Plamberger S., Horner D. (2022 May 10). Sample buffer containing guanidine-hydrochloride combines biological safety and RNA preservation for SARS-CoV-2 molecular diagnostics. *Diagnostics*.

[55] Welch S. R., Davies K. A., Buczkowski H. (2020). Analysis of inactivation of SARS-CoV-2 by specimen transport media, nucleic acid extraction reagents, detergents, and fixatives. *Journal of Clinical Microbiology*.

[56] WHO (2020). Laboratory testing for Coronavirus disease (COVID-19) in suspected human cases. https://apps.who.int/iris/rest/bitstreams/1272156/retrieve.

[57] Freire-Paspuel B., Vega-Mariño P., Velez A. (2020). Cotton-tipped plastic swabs for SARS-CoV-2 RT-qPCR diagnosis to prevent supply shortages. *Frontiers in Cellular and Infection Microbiology*.

[58] Nairz M., Bellmann-Weiler R., Ladstätter M. (2021). Overcoming limitations in the availability of swabs systems used for SARS-CoV-2 laboratory diagnostics. *Scientific Reports*.

[59] Luinstra K., Petrich A., Castriciano S. (2011). Evaluation and clinical validation of an alcohol-based transport medium for preservation and inactivation of respiratory viruses. *Journal of Clinical Microbiology*.

[60] Dehbandi R., Zazouli M. A. (2020). Stability of SARS-CoV-2 in different environmental conditions. *The Lancet Microbe*.

[61] BMKG (2021). Tren Suhu. https://www.bmkg.go.id/iklim/?p=tren-suhu.

