# Supplementary material for: Evaluation and Clinical Validation of Guanidine-Based Inactivation Transport Medium for Preservation of SARS-CoV-2
Source: Adv Pharmacol Pharm Sci. 2022 Jul 21;2022:1677621. doi: 10.1155/2022/1677621 (PMC9301760; doi:10.1155/2022/1677621)
Supplement: Supplementary Materials — Additional file APPENDIX: (I) Appendix 1. RT-PCR results of COVID-19 samples stored in VITPAD® ITM at room temperature (±25°C) for different storage days. (II) Appendix 2. RT-PCR results of COVID-19 samples in VITPAD® ITM, extracted with and without lysis buffer. (III) Appendix 3. RT-PCR results of COVID-19 samples stored in VITPAD® ITM, incubated at 40°C for 3 hours. (IV) Appendix 4. Receiver operating characteristic (ROC) curve statistical analysis. (V) Appendix 5. t-test for the safety of VITPAD® ITM. (VI) Appendix 6. t-test for the resistance of VITPAD® ITM. [file 1677621.f1.docx]

**APPENDIX**

Appendix 1. RT-PCR results of COVID-19 samples stored in VITPAD^®^ ITM at room temperature (±25^o^C) for different storage days.

| **No.** | **Code** | **Day 0** | | | | **Day 4** | | | | **Day 8** | | | | **Day 10** | | | | **Day 18** | | | |
| --- | --- | --- | --- | --- | --- | --- | --- | --- | --- | --- | --- | --- | --- | --- | --- | --- | --- | --- | --- | --- | --- |
|  |  | **ORF1ab** | **N-Gene** | **E-Gene** | **INTP.** | **ORF1ab** | **N-Gene** | **E-Gene** | **INTP.** | **ORF1ab** | **N-Gene** | **E-Gene** | **INTP.** | **ORF1ab** | **N-Gene** | **E-Gene** | **INTP.** | **ORF1ab** | **N-Gene** | **E-Gene** | **INTP.** |
| 1 | **VS-1** | **20.75** | **19.78** | **22.04** | **P** | **22.52** | **21.05** | **22.32** | **P** | **22.31** | **20.52** | **22.68** | **P** | **23.70** | **21.07** | **23.90** | **P** | **20.67** | **19.31** | **21.26** | **P** |
| 2 | **VS-2** | **20.20** | **19.72** | **21.54** | **P** | **27.10** | **25.28** | **26.57** | **P** | **24.68** | **22.77** | **25.37** | **P** | **28.32** | **25.28** | **28.26** | **P** | **26.87** | **24.87** | **27.02** | **P** |
| 3 | **VS-3** | **26.66** | **27.85** | **26.59** | **P** | **26.95** | **25.03** | **26.27** | **P** | **25.72** | **23.65** | **26.28** | **P** | **27.23** | **24.24** | **26.97** | **P** | **25.75** | **24.04** | **26.03** | **P** |
| 4 | **VS-4** | **29.80** | **30.62** | **31.13** | **P** | **25.96** | **23.99** | **25.23** | **P** | **25.71** | **23.54** | **26.15** | **P** | **28.27** | **25.11** | **28.03** | **P** | **24.78** | **22.85** | **25.16** | **P** |
| 5 | **VS-5** | **22.51** | **23.49** | **23.89** | **P** | **19.26** | **18.15** | **19.14** | **P** | **16.56** | **15.62** | **17.50** | **P** | **19.34** | **17.52** | **19.91** | **P** | **17.04** | **16.38** | **17.87** | **P** |
| 6 | **VS-6** | **24.77** | **25.28** | **26.08** | **P** | **18.93** | **17.14** | **18.91** | **P** | **17.52** | **15.78** | **18.31** | **P** | **20.72** | **18.26** | **20.92** | **P** | **18.98** | **16.98** | **19.46** | **P** |
| 7 | **VS-7** | **25.96** | **27.23** | **26.60** | **P** | **25.43** | **23.40** | **25.21** | **P** | **25.12** | **22.82** | **25.72** | **P** | **28.71** | **25.40** | **28.47** | **P** | **25.29** | **23.29** | **25.80** | **P** |
| 8 | **VS-8** | **21.85** | **23.59** | **23.31** | **P** | **28.56** | **27.34** | **28.72** | **P** | **28.42** | **26.75** | **29.01** | **P** | **29.87** | **28.14** | **31.54** | **P** | **30.76** | **28.78** | **31.18** | **P** |
| 9 | **VS-9** | **25.36** | **25.12** | **25.89** | **P** | **21.38** | **19.26** | **21.14** | **P** | **19.84** | **17.69** | **20.47** | **P** | **21.80** | **19.40** | **22.18** | **P** | **20.55** | **18.53** | **21.27** | **P** |
| 10 | **VS-10** | **18.35** | **19.10** | **17.90** | **P** | **22.38** | **20.69** | **22.25** | **P** | **21.82** | **19.89** | **22.45** | **P** | **24.12** | **21.81** | **24.08** | **P** | **23.03** | **21.25** | **23.27** | **P** |
| 11 | **VS-11** | **25.18** | **24.16** | **25.70** | **P** | **27.31** | **25.68** | **27.38** | **P** | **26.61** | **24.48** | **26.90** | **P** | **28.52** | **25.71** | **28.45** | **P** | **26.95** | **25.38** | **27.49** | **P** |
| 12 | **VS-12** | **15.72** | **13.32** | **15.44** | **P** | **18.83** | **17.97** | **19.14** | **P** | **17.47** | **16.51** | **18.56** | **P** | **20.07** | **18.25** | **20.39** | **P** | **17.66** | **18.12** | **17.70** | **P** |
| 13 | **VS-13** | **29.97** | **28.14** | **30.12** | **P** | **27.85** | **25.89** | **27.32** | **P** | **27.52** | **25.11** | **27.98** | **P** | **30.80** | **28.10** | **30.29** | **P** | **26.23** | **24.48** | **26.14** | **P** |
| 14 | **VS-14** | **27.54** | **27.30** | **27.01** | **P** | **23.31** | **21.71** | **23.26** | **P** | **21.59** | **20.08** | **22.43** | **P** | **22.86** | **21.03** | **22.97** | **P** | **21.84** | **20.45** | **22.41** | **P** |
| 15 | **VS-15** | **16.74** | **18.46** | **17.51** | **P** | **26.97** | **26.05** | **27.14** | **P** | **23.85** | **22.70** | **25.08** | **P** | **27.69** | **25.16** | **28.18** | **P** | **24.41** | **23.48** | **25.48** | **P** |
| 16 | **VS-16** | **25.95** | **26.20** | **26.06** | **P** | **33.22** | **30.18** |  | **P** |  | **30.51** | **32.28** | **P** | **33.07** | **29.14** | **32.49** | **P** | **30.92** | **29.56** | **31.22** | **P** |
| 17 | **VS-17** | **29.78** | **31.17** | **30.76** | **P** | **21.43** | **19.94** | **21.41** | **P** | **20.27** | **18.31** | **21.19** | **P** | **24.97** | **22.42** | **24.99** | **P** | **21.30** | **19.62** | **21.85** | **P** |
| 18 | **VS-18** | **29.66** | **30.69** | **30.98** | **P** | **30.04** | **28.15** | **29.47** | **P** | **29.68** | **28.44** | **30.61** | **P** | **31.31** | **29.19** | **32.09** | **P** | **29.28** | **28.18** | **29.68** | **P** |
| 19 | **VS-19** | **26.34** | **27.85** | **26.61** | **P** | **25.19** | **23.51** | **24.88** | **P** | **25.07** | **22.80** | **25.71** | **P** | **27.33** | **24.67** | **27.27** | **P** | **25.94** | **23.95** | **26.38** | **P** |
| 20 | **VS-20** | **18.09** | **19.09** | **18.43** | **P** | **20.13** | **19.04** | **20.26** | **P** | **19.53** | **18.35** | **20.86** | **P** | **21.54** | **19.58** | **22.07** | **P** | **19.95** | **19.09** | **20.93** | **P** |
| 21 | VS-21 |  |  |  | N |  |  |  | N |  |  |  | N |  |  |  | N |  |  |  | N |
| 22 | VS-22 |  |  |  | N |  |  |  | N |  |  |  | N |  |  |  | N |  |  |  | N |
| 23 | VS-23 |  |  |  | N | **32.87** | **29.93** |  | **P** |  |  |  | N |  |  |  | N |  |  |  | N |
| 24 | VS-24 |  |  |  | N |  |  |  | N |  |  |  | N |  |  |  | N |  |  |  | N |
| 25 | VS-25 |  |  |  | N |  |  |  | N |  |  |  | N |  |  |  | N |  |  |  | N |
| 26 | VS-26 |  |  |  | N |  |  |  | N |  |  |  | N |  |  |  | N |  |  |  | N |
| 27 | VS-27 |  |  |  | N |  |  |  | N |  |  |  | N |  |  |  | N |  |  |  | N |
| 28 | VS-28 |  |  |  | N |  |  |  | N |  |  |  | N |  |  |  | N |  |  |  | N |
| 29 | VS-29 |  |  |  | N |  |  |  | N |  |  |  | N |  |  |  | N |  |  |  | N |
| 30 | VS-30 |  |  |  | N |  |  |  | N |  |  |  | N |  |  |  | N |  |  |  | N |

*INTP-Interpretation. P-Positive. N-Negative. Target Gene-ORF1ab, N-Gene, E-Gene.

Appendix 2. RT-PCR results of COVID-19 samples stored in VITPAD^®^ ITM, extracted with and without lysis buffer.

| **No.** | **Code** | **With Lysis Buffer** | | | | **Without Lysis Buffer** | | | |
| --- | --- | --- | --- | --- | --- | --- | --- | --- | --- |
|  |  | **ORF1ab** | **N-Gene** | **E-Gene** | **INTP.** | **ORF1ab** | **N-Gene** | **E-Gene** | **INTP.** |
| **1** | **VK-1** | **24.87** | **22.34** | **24.39** | **P** | **26.00** | **23.72** | **25.60** | **P** |
| **2** | **VK-2** | **27.59** | **26.71** | **27.69** | **P** | **28.69** | **27.92** | **28.83** | **P** |
| **3** | **VK-3** | **19.60** | **18.89** | **20.09** | **P** | **19.51** | **19.17** | **20.18** | **P** |
| **4** | **VK-4** | **16.44** | **15.77** | **17.04** | **P** | **17.28** | **16.47** | **17.96** | **P** |
| **5** | **VK-5** | **23.90** | **23.94** | **24.60** | **P** | **25.24** | **25.04** | **25.89** | **P** |
| **6** | **VK-6** | **25.72** | **24.52** | **26.09** | **P** | **26.00** | **24.91** | **26.38** | **P** |
| **7** | **VK-7** | **22.27** | **20.91** | **22.56** | **P** | **22.97** | **21.40** | **23.20** | **P** |
| **8** | **VK-8** | **29.48** | **28.52** | **29.46** | **P** | **29.93** | **28.96** | **30.18** | **P** |
| **9** | **VK-9** | **21.43** | **20.19** | **21.84** | **P** | **22.41** | **20.92** | **22.78** | **P** |
| **10** | **VK-10** | **25.47** | **25.23** | **26.30** | **P** | **26.77** | **26.52** | **27.52** | **P** |
| **11** | **VK-11** | **18.08** | **19.27** | **18.74** | **P** | **19.75** | **20.90** | **20.27** | **P** |
| **12** | **VK-12** | **23.06** | **21.92** | **23.35** | **P** | **23.95** | **22.28** | **23.80** | **P** |
| **13** | **VK-13** | **29.88** | **30.68** | **31.01** | **P** | **30.60** | **30.61** | **31.63** | **P** |
| **14** | **VK-14** | **25.60** | **26.91** | **26.61** | **P** | **25.93** | **27.01** | **26.68** | **P** |
| **15** | **VK-15** | **24.45** | **23.79** | **24.56** | **P** | **25.17** | **24.74** | **25.60** | **P** |
| **16** | **VK-16** | **29.76** | **28.37** | **29.10** | **P** | **31.29** | **29.33** | **30.72** | **P** |
| **17** | **VK-17** | **23.41** | **22.24** | **23.77** | **P** | **24.05** | **22.73** | **24.56** | **P** |
| **18** | **VK-18** | **20.73** | **20.64** | **21.75** | **P** | **20.71** | **20.46** | **21.59** | **P** |
| **19** | **VK-19** | **28.96** | **27.83** | **28.96** | **P** | **29.62** | **28.33** | **29.66** | **P** |
| **20** | **VK-20** | **34.79** | **33.38** | **32.52** | **P** | **36.94** | **33.44** | **36.92** | **P** |
| **21** | **VK-21** | **31.18** | **29.55** | **28.55** | **P** | **31.35** | **28.97** | **28.77** | **P** |
| **22** | **VK-22** | **32.08** | **30.88** | **29.74** | **P** | **30.47** | **27.55** | **28.53** | **P** |
| 23 | VK-23 |  |  |  | N |  |  |  | N |
| 24 | VK-24 |  |  |  | N |  |  |  | N |
| 25 | VK-25 |  |  |  | N |  |  |  | N |
| 26 | VK-26 |  |  |  | N |  |  |  | N |
| 27 | VK-27 |  |  |  | N |  |  |  | N |
| 28 | VK-28 |  |  |  | N |  |  |  | N |
| 29 | VK-29 |  |  |  | N |  |  |  | N |
| 30 | VK-30 |  |  |  | N |  |  |  | N |
| 31 | VK-31 |  |  |  | N |  |  |  | N |
| 32 | VK-32 |  |  |  | N |  |  |  | N |
| 33 | VK-33 |  |  |  | N |  |  |  | N |
| 34 | VK-34 |  |  |  | N |  |  |  | N |
| 35 | VK-35 |  |  |  | N |  |  |  | N |
| 36 | VK-36 |  |  |  | N |  |  |  | N |
| 37 | VK-37 |  |  |  | N |  |  |  | N |
| 38 | VK-38 |  |  |  | N |  |  |  | N |

*INTP-Interpretation. P-Positive. N-Negative. Target Gene-ORF1ab, N-Gene, E-Gene.

Appendix 3. RT-PCR results of COVID-19 samples stored in VITPAD^®^ ITM, incubated at 40^o^C for 3 hours.

| **No.** | **Code** | **40^o^C (3 hours)** | | | | **Room temperature (± 25^o^C)** | | | |
| --- | --- | --- | --- | --- | --- | --- | --- | --- | --- |
|  |  | **ORF1ab** | **N-Gene** | **Internal Control** | **INTPN.** | **ORF1ab** | **N-Gene** | **Internal Control** | **INTPN.** |
| **1** | **VP-1** | **31.37** | **30.09** | **26.63** | **P** | **33.6** | **32.98** | **23.98** | **P** |
| **2** | **VP-2** | **30.52** | **29.61** | **23.29** | **P** | **28.99** | **28.48** | **23.19** | **P** |
| **3** | **VP-3** | **25.77** | **24.75** | **22.38** | **P** | **25.14** | **23.72** | **21.76** | **P** |
| **4** | **VP-4** | **26.66** | **25.37** | **23.44** | **P** | **26.68** | **25.28** | **23.53** | **P** |
| **5** | **VP-5** | **34.43** | **33.10** | **23.40** | **P** | **32.73** | **31.72** | **21.84** | **P** |
| **6** | **VP-6** | **25.82** | **25.53** | **21.76** | **P** | **25.82** | **25.18** | **21.46** | **P** |
| **7** | **VP-7** | **22.95** | **22.21** | **23.55** | **P** | **28.92** | **28.88** | **22.94** | **P** |
| **8** | **VP-8** | **30.75** | **28.87** | **23.46** | **P** | **27.7** | **27.02** | **22.76** | **P** |
| **9** | **VP-9** | **16.76** | **15.28** | **21.94** | **P** | **21.45** | **20.92** | **23.00** | **P** |
| **10** | **VP-10** | **31.09** | **29.88** | **23.06** | **P** | **32.96** | **32.52** | **23.21** | **P** |
| **11** | **VP-11** | **15.35** | **14.84** | **22.91** | **P** | **14.19** | **14.58** | **23.17** | **P** |
| **12** | **V0-12** | **17.99** | **17.98** | **23.41** | **P** | **17.38** | **17.43** | **22.84** | **P** |

*INTP-Interpretation. P-Positive. N-Negative. Target Gene-ORF1ab, N-Gene, Internal Control.

Appendix 4. Receiver Operating Characteristic (ROC) curve statistical analysis.

| **AREA UNDER THE CURVE** | | | | | | | | | |
| --- | --- | --- | --- | --- | --- | --- | --- | --- | --- |
| IC CT value Sansure of VITPAD^®^ ITM | | | | | IC CT value Sansure of NEST ITM | | | | |
| Area | Std. Error^a^ | Asymptotic Sig. ^b^ | Asymptotic 95% Confidence Interval | | Area | Std. Error^a^ | Asymptotic Sig. ^b^ | Asymptotic 95% Confidence Interval | |
|  |  |  | Lower Bound | Upper Bound |  |  |  | Lower Bound | Upper Bound |
| 0.550 | 0.077 | 0.502 | 0.399 | 0.700 | 0.731 | 0.071 | 0.004 | 0.591 | 0.871 |

The test result variables: IC Sansure has at least one tie between the positive actual state group and the negative actual state group. Statistics may be biased.

Appendix 5. t-test for the safety of VITPAD^®^ ITM.

| **Unpaired t test** | **ORF1ab** | **N-Gene** | **E-Gene** |
| --- | --- | --- | --- |
| P value | 0.615 | 0.761 | 0.510 |
| Significantly different (P < 0.05)? | No | No | No |
| t | 0.507 | 0.306 | 0.660 |
| df | 42 | 42 | 42 |
| 95% confidence interval | -2.153 to 3.596 | -2.265 to 3.074 | -1.732 to 3.416 |
| R squared (eta squared) | 0.006 | 0.002 | 0.010 |

Appendix 6. t-test for the resistance test of VITPAD^®^ ITM.

| **Unpaired t test** | **ORF1ab** | **N-Gene** | **Internal Control** |
| --- | --- | --- | --- |
| P value | 0.840 | 0.700 | 0.270 |
| Significantly different (P < 0.05)? | No | No | No |
| t | 0.201 | 0.384 | 1.133 |
| df | 22 | 22 | 22 |
| 95% confidence interval | -5,763 to 4,747 | -5,977 to 4,111 | -0,3879 to 1,321 |
| R squared (eta squared) | 0.002 | 0.007 | 0.055 |
